# Assessing gender disparities in farmers’ access and use of climate-smart agriculture in Southern Tanzania

**DOI:** 10.1007/s43621-025-01150-8

**Published:** 2025-04-28

**Authors:** Eileen Bogweh Nchanji, Agness Ndunguru, Catherine Kabungo, Adolph Katunzi, Victor Nyamolo, Fredrick Ochieng Ouya, Mercy Mutua, Boaz Waswa, Cosmas Kweyu Lutomia

**Affiliations:** 1 International Center for Tropical Agriculture, Nairobi, Kenya; 2 Tanzania Agricultural Research Institute (TARI), Uyole, Tanzania; 3 Egerton University, Nakuru, Kenya

**Keywords:** Gender, Climate-smart agricultural technologies, Food security, Adaptation, Decision-making, Youth

## Abstract

The importance of common bean in Tanzania is increasingly challenged by climate change, which increases women's vulnerability and undermines the contribution of the crop to food security and rural livelihoods. This study assessed gender differences in the use of climate-smart agriculture technologies and practices among bean farmers in Tanzania. A multi-stage sampling procedure was used to collect data from 364 smallholder bean farmers. Descriptive statistics and a multivariate probit model were employed to analyse the determinants of farmers’ adoption of climate-smart agricultural technologies and practices in common bean production. Results revealed that men dominated climate-adaptation decision-making processes at the household level because of their ownership and control over access to land, and access to agricultural support services. Older men farmers demonstrated a positive and significantly higher likelihood of adopting improved seeds (β = 0.026; *p* < 0.01), signifying they possess greater accumulated knowledge and wealth compared to women farmers and youths. Women farmers also had lower levels of education with fewer technological access contributing to their low uptake of climate-smart technologies, aggravating their vulnerability to climate change. Enhancing inclusive gender access to land and group-based approaches to information dissemination, and capacity building, would be relevant in enabling men, women, and young farmers to improve their adaptive and resilience capacities to climate change. Gender dynamics should be considered in designing climate-smart agriculture policies and implementation of climate-smart agriculture  programs and policies to improve farmers’ resilience to climate change.

## Introduction

Agriculture is critical to the economic growth of Sub-Saharan African (SSA) countries but is negatively affected by climate change [1]. Droughts, erratic rains, flooding, and crop pests and diseases experienced  across the African continent negatively impact the livelihoods of millions of people, especially women. Specifically,  climate change adversely affect agricultural production [2] and widens existing gender inequalities in agri-food systems [3]. In the last two decades, frequent droughts and erratic rainfall have reduced crop productivity among farming households with limited diversification options [2]. Smallholder farmers and a majority of people  who live below the poverty line are the most affected because of their heavy reliance on rain-fed agriculture as their primary source of livelihood [4].

In the last two decades, frequent droughts and erratic rainfall have reduced crop productivity among farming households with limited diversification options [2]. Smallholder farmers’ vulnerability to climate change and weather variability is further worsened by pre-existing conditions, including low access to markets, weak institutional support and policy, low technology adoption, and poverty [2, 5–7]. Smallholder farmers’ capacity to adapt to climate change and weather variability is thus undermined [8], resulting in poor harvests, low yields, malnutrition, poverty, and high dependency on food imports and aid. Women smallholder farmers are more vulnerable than men smallholder farmers because they represent the majority of the world’s poor and are proportionally more dependent on threatened natural resources [9, 10].

In Tanzania, climate change has caused food shortages and resulted in decreased household income among smallholder farmers [11]. The impacts of climate change are felt across the country because agriculture employs about 80% of Tanzania’s population directly or indirectly [12] with women providing about 50% of labour and low youth involvement in agriculture [13]. Nonetheless, the impacts of climatic shocks are skewed with women being the most vulnerable group in Tanzania [14]. With more women depending on agriculture as the primary source of income than men, they are often the most affected by reduced agricultural output induced by climate change [15]. Climate change and variability are increasingly becoming the greatest threat to tackling poverty, affecting many aspects of development work, and worsening existing gender inequality [16].

Smallholder farmers in Tanzania, especially women cultivate relatively small pieces of land and often lack access to reliable irrigation. The majority of them also lack access to sufficient labour, inputs, and financial support [17]. Climatic shocks exacerbate the existing challenges faced by smallholder farmers, leading to the continuous devastation of their livelihoods, and consequently accentuating poverty [18]. Climate change has undermined the ability of Tanzania to achieve the Sustainable Development Goals [19]. Floods, erratic rains, and droughts negatively impact the realization of actions to end poverty (SDG 1), zero hunger (SDG 2), gender equality (SDG 5), and climate action (SDG 13). Low production of staple grains caused by recurrent droughts and pests and diseases reduce sustained availability and accessibility to food which exacerbates existing food and nutrition challenges, disproportionately affecting women [20].

Climate-smart agriculture is part of the climate action strategies that have the potential to simultaneously enhance food security and reduce poverty and gender disparities [21]. Nonetheless, the use of climate-smart agriculture such as conservation agriculture, agro-forestry, integrated pest management, rainwater harvesting, improved seed innovations, and fertilizer varies across agro-ecologies. The social, economic, agro-ecological, policy and institutional aspects influence the use of climate-smart technologies and practices in different contexts [22, 23]. The inherent gender gaps in agriculture affect access and utilization of climate-smart agriculture. The considerably low capacity of women to adapt to climate change and variability is attributed to their restricted access to resources such as land and finance, low technical ability because of low access to information, extension, and training among other vulnerabilities in agriculture [24]. Social networks have played a critical role among smallholder farmers in Tanzania to build resilience against climatic shocks [25]. They have enhanced access to climate-smart agricultural training, which has enabled smallholder farmers to develop effective mechanisms to offset the adverse impacts of climate change and variability in their farming communities.

Therefore, strengthening the capacity of women farmers and youth to uptake climate-smart agriculture is a crucial step towards bridging the gender gaps in agriculture in Tanzania and other countries in the region. According to the African Union’s classifications, a youth is any individual aged between 15 and 35 years [26]. Youth are critical in advancing climate-smart agriculture (CSA) due to their adaptability to new technologies and openness to innovation. They infuse ingenuity and innovativeness in agriculture, helping address multifaceted challenges of food insecurity, climate change adaptation, and sustainable rural development. As future stewards of agricultural lands, youth engagement ensures the long-term sustainability of farming, creating a generation of environmentally conscious farmers. Their openness to practices like soil conservation, water management, and agroforestry allows them to act as change agents and advocate for climate-resilient farming within their communities. However, despite the inherent gender gaps in agriculture, few studies have focused on how access and use of these technologies/practices vary by gender and age. Additionally, gender differences in access and use of climate-smart agriculture have not been studied in detail for bean farmers in Mbeya rural district and Mbozi district, the major common bean productions hubs in Tanzania. Consequently, this study investigated the role of gender in the uptake of climate-smart agricultural practices in Tanzania.

## Conceptual framework

Climate-smart agriculture encompasses various practices and technologies to mitigate effects of climate change. Several contextual factors categorized as human capital, physical capital, social capital, financial capital, natural capital and demographic factors influence the use of climate-smart technologies and practices, especially among vulnerable communities [27]. Human capital, including education, is a critical factor that determines farmers’ ability to cope and adapt to the impacts of climate change. Education level of farmers is cited as an important human capital factor influencing the adoption of climate-smart agriculture [28] with women often less educated than men. Educated farmers make better decisions about the adoption of new technologies because of their ability to access, understand, and use technical information related to climate change adaptation [23]. Therefore, the study anticipated that human capital would influence the use of climate-smart agriculture.

Physical capital, such as communication assets for instance mobile phones and radio, influence farmers’ access to agricultural information from digital social networks in real-time [25]. Ownership and use of communication assets differ by gender with women being disadvantaged than men in terms of access to climate information and advisory services via digital platforms [29, 30]. 

Local community institutions are important in climate change management discourse and are platforms for external support and interventions in climate change actions because they influence resource mobilization, creating networks, information gathering and sharing, skills development, and capacity building of farmers [31, 32]. They mediate farmers’ access to agro-dealers, insurance, and credit which are critical services and resources for adoption of climate-smart agriculture.  However, participation in local institutions is not gender neutral as women participation is constrained by the double burden of domestic and farm work, low input in group decisions, and representation in group governance  [33, 34]. Natural capital such as land and water resources also influence the uptake of climate-smart technologies and practices. Land and land use decision-making matters in farm-level climate change adaptation processes [35, 36]. In sub-Saharan Africa, sociocultural norms designate men as the primary landowners and land use decision-makers, while women and youth have limited access and decision-making power over land  [37].

**Fig. 1:**
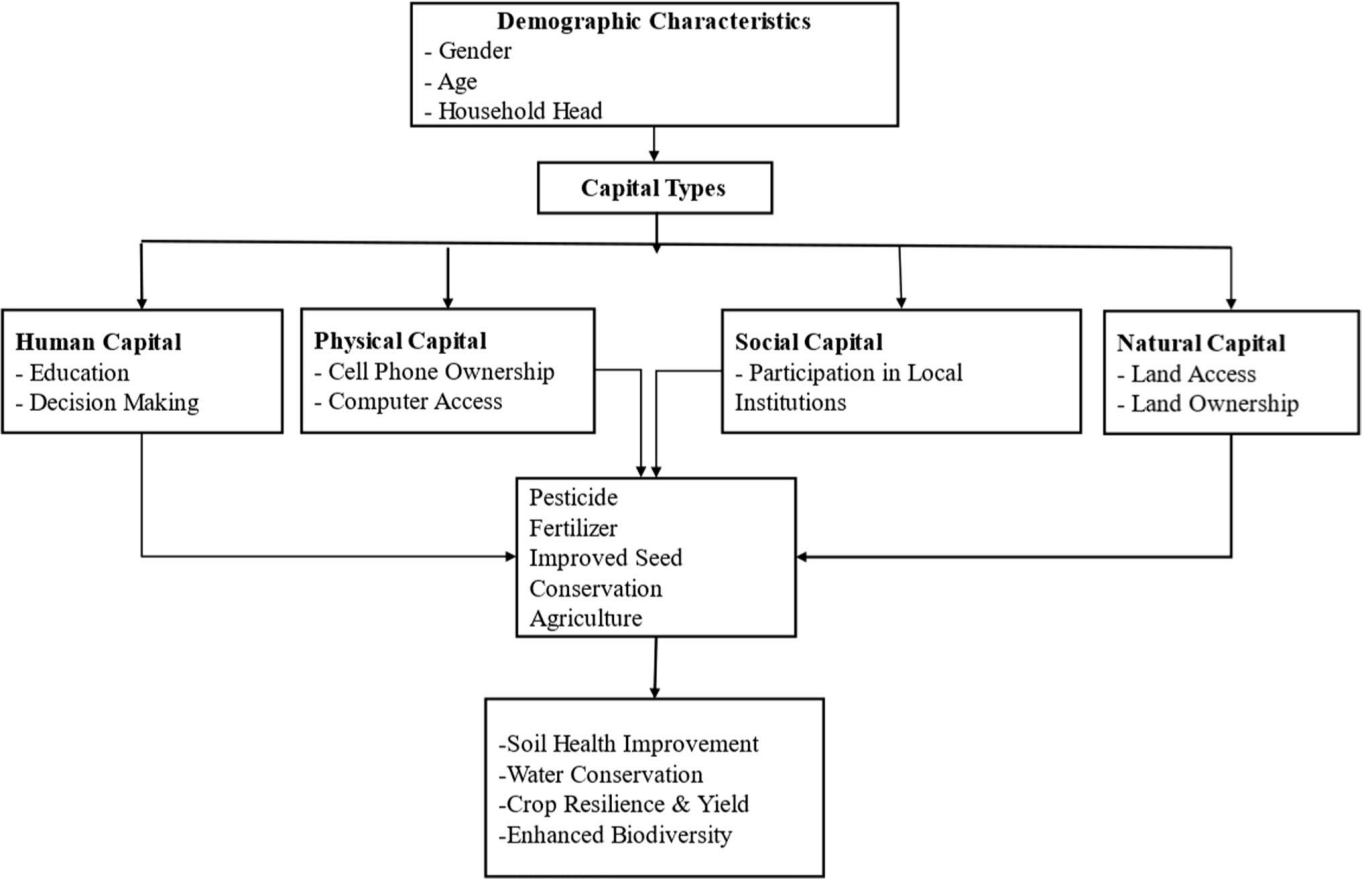
Conceptual framework

Additionally,  individual and demographic characteristics such as age, household headship, gender, primary occupation, marital status, household type and farm-level factors are important determinants of technology adoption.  Therefore, the study conceptualizes that individual demographic characteristics, human, social, and natural capital, and access to support services (extension and training) influence access and use of climate-smart technologies and practices as shown in Fig. 1. However, the role of financial capital was not considered because of data limitations.

## Methodology

### Study area

The study was conducted in six wards in the Mbeya rural and Mbozi districts in the Southern Highlands zone of Tanzanian. Farming is the primary source of livelihood in the two districts that are characterized by bimodal rainfall pattern, with the main season starting in October to May and short season occurring between June through September [38]. Mbeya rural and Mbozi districts are in the Southern agro-ecological region that is characterized by cooler highlands, mid-attitude, and lowland climatic conditions. The rainfall amounts in the two regions ranges from 650 to 2600 mm per annum with seasonal and annual variations [38]. Temperatures vary depending on altitude but generally range from 16 to 30 °C. The agro-ecological conditions favour production of staple foods such as maize, beans, rice, potato, and wheat. In recent decades, climate change and variability has greatly affected crop production in these two districts. Water availability is increasingly becoming a major crop production constraint because of increasing frequency of drought. The deteriorating water quality, loss of biodiversity, and a significant decline in agricultural production are some of the consequences of the adverse impacts of climate change and variability already witnessed in these districts [39].

### Sampling procedure and sample size determination

Production systems vary across districts, supporting diverse crops and livestock. The districts have been intervention areas of several private and public agricultural development projects focusing on food crops such as maize and common bean [40]. Interventions in the common bean value chain  involve  several organizations that partner with the Tanzania Agricultural Research Institute (TARI). In addition, several farmer networks with diverse characteristics have been used as outreach platforms, making bean growing population large and heterogeneous.

A multi-stage sampling procedure was adopted in this study where the Southern highland region was purposively selected as it is the major common beans production area in Tanzania. Common bean has been traditionally considered as a women’s crop in the region. Consequently, gender transformative approaches are increasingly being integrated into common bean into, seed systems, and market interventions. Therefore, this study targeted smallholder farmers involved in bean production in Mbeya rural and Mbozi districts. Three wards were randomly selected from each district to enhance representation: Mshewe, Idiwili and Bonde Lasongwe in Mbeya rural district and Magamba, Isansa and Itumpi in Mbozi district. The third step involved random selection of one village in each ward, hence a total of 6 villages were sampled. Lists of registered common bean farmers from the selected villages were obtained from TARI officers and used as the sampling frame.

With the population from the six sampled villages known, the sample size was determined by Yamane [41] formula as below i.e.,

1 $$n=\frac{N}{1+N{(e)}^{2}}$$

where: $n$ is the sample size; $N$ is the population size (number of bean farmers registered with TARI); and $e$ is the desired level of precision.

This study sampled a total of 360 households. Following the evenly proportional distribution of bean farmers across the selected villages, 60 respondents were sampled from each of the 6 villages, where systematic random sampling was employed in every selected village and replacements were done using the same method. Since more is preferred to less and for improved precision, an increase of 2% was done to cater for incomplete or invalid responses, non-responses, or any unexpected issue hence the target was increased to approximately 368 respondents. The achieved sample was 364 men and women respondents.

### Data collection

The study employed a quantitative research design, in collecting data from bean growers in the two districts. The tool was co-developed by all stakeholders and pre-tested for consistency and validity before administration to selected farmers. The questionnaire had five sections that collected diverse information from respondents. The first section collected household location and sociodemographic details, including age, sex, education, household headship, and occupation. The second section of the survey tool collected data on land ownership, access, and allocation to bean production across seasons, and decision-making about bean production. The third section of the questionnaire covered types of seed and bean varieties planted by farmers and bean production practices. Section four of the questionnaire collected data on production constraints, while section five collected data on farmers’ access to information on bean production, technologies, and marketing. The survey tool was programmed in ODK and administered to farmers by trained enumerators from the Tanzania Agricultural Research Institute (TARI). 

### Data analysis

#### Descriptive method

Mean and standard deviation were used to describe numeric continuous variables, while categorical variables were cross-tabulated to obtain frequencies and percentages of farmers’ responses. Analysis of variance (ANOVA) and chi-square test of independence were performed to measure systematic  difference in the distribution of continuous and categorical variables by gender and age, respectively.

#### Econometric model specification

The role of sociodemographic, farm level and institutional factors in explaining the use of climate-smart agriculture in the study area was estimated using a multivariate probit model. Unlike univariate probit or logit models that rely on a single response of either yes or no if a farmer use climate-smart agricultural technology or practice, a multivariate probit approach was considered because it allows simultaneous estimation of regression equations, in this case, the simultaneous use of improved bean seed, integrated pests management, fertilizer, and conservation agriculture without any expectations a priori [42]. Simultaneous estimation of the four regression equations allows the exploitation of interrelationships among climate-smart agriculture practices and technologies based on the assertion that farmers adopt agriculture technologies as bundles rather than isolated practices [42].

The multivariate probit model for the use of climate-smart agricultural technologies and practices as a set of binary dependent variables is written as follows:

$${y}_{m}^{*}= {\beta }_{m}{\prime}{X}_{m}+ {\varepsilon }_{m} , n=1,\dots \dots \dots \dots \dots .M$$

2 $${y}_{m}=\left\{\begin{array}{c}if \; {y}_{m}^{*}>0 \\ 0\, otherwise\end{array}\right.$$

Let ${y}_{m}$ denotes a random variable a signed values of (1, 2, 3, and 4) for positive integers, representing four climate-smart agricultural technologies. X denotes a set of explanatory variables including sociodemographic, economic, farm, digital technology, and institutional characteristics. ${\varepsilon }_{m}$ is the disturbance term, Where ${\beta }_{m}{\prime}$ are parameters to be estimated and ${y}_{m}^{*}$ is the latent variable and $\varepsilon_{in} ,n = 1,.........N$, are the error terms distributed as multivariate normal, with each having zero mean and variance–covariance matrix [43]. The off diagonals of the regression models are collections between the four equations on which the assumption of independence of the equations is made.

## Results and discussions

### Descriptive results

Table 1 presents the socio-demographic characteristics of the study respondents, disaggregated by sex. The results explore differences between male and female respondents across various socioeconomic characteristics of adult and youth farmers. The findings indicate significant differences in socioeconomic characteristics, primarily manifested among adult farmers rather than youth. Only age shows a notable difference between male and female youth, suggesting that further disaggregation of youth by sex may not yield meaningful insights into the socioeconomic dynamics of this group. Focusing on youth as a cohesive demographic thus allows for a more comprehensive understanding of the collective challenges and opportunities they face, which might be overshadowed if analyzed through a gender lens that may not reveal significant distinctions [44, 45]. Additionally, youth in agricultural contexts often share common experiences related to education, access to resources, and economic opportunities that are more indicative of their collective situation rather than individual gender differences [46]. Therefore, maintaining the youth demographic as a single category ensures targeted analysis towards the unique circumstances influencing this group, thereby avoiding unnecessary complexity without substantial justification for gender-based differentiation. This approach emphasizes the importance of understanding youth as a distinct segment within the agricultural sector to inform tailored interventions and policies that address their specific needs.

**Table 1:** Socio-economic characteristics of youth and adult farmers by sex

Variable		Youth farmers (N = 132)			Adult farmers (N = 225)		
		Male (n = 61)	Female (n = 71)	*p*-value	Male (n = 115)	Female (n = 110)	*p*-value
Age		29.84	28.48	0.014	49.14	47.98	0.110
Marstat				0.715			0.000
Never married		6.00	1.32		0.87	0.89	
Married—polygamous		3.00	21.05		27.83	23.21	
Married—monogamous		52.00	67.11		69.57	45.54	
Widowed		0.00	1.32		1.74	24.11	
Divorced/separated		0.00	0.00		0.00	6.25	
Presence on agriculture social media platform (%)		8.20	5.26	0.975	2.61	0.91	0.004
Education level				0.234			0.001
No formal education		9.91	9.67		2.61	18.75	
Primary level		57.37	78.69		87.83	75.00	
Secondary level (0)		34.42	36.62		9.57	5.36	
Tertiary		0.82	0.28		0.00	0.89	
Average acres of land accessed		3.43	3.33	0.219	5.83	3.78	0.001
Distance to Agrodealer		17.19	18.92	0.975	16.47	17.65	0.441
Own cellphone		96.72	88.16	0.612	95.65	83.93	0.003
Occupation				0.215			0.777
Crop farming		78.69	84.21		83.48	83.93	
Formal employment (with salary)		21.31	15.79		15.65	14.29	
Group membership		39.34	40.79	0.124	48.70	39.29	0.153

Table 2 presents the probability value (*p*-value) column of the ANOVA test result for the age of respondents (a continuous variable), and the chi-square test for categorical variables (gender, relationship to household head, education level, main occupation, marital status, and household type). The results showed that the average age of respondents was 41 years and that of men were averagely 49 years, women 48 years, and youths 29 years (*p* < 0.01). This finding suggests that older individuals are more likely to engage in farming thus reinforcing agriculture as the main source of livelihood in Tanzanian rural areas. The findings further revealed that 64% of respondents were household heads with a significantly higher percentage of men (100%) reporting being household heads than women (39%) and youths (52%) (*p* < 0.01). These findings reflect the deep-seated  sociocultural context in sub-Saharan Africa that designate household headship to male household members [47].

**Table 2:** Sociodemographic characteristics of respondents by gender

Variable	Overall (N = 357)	Youths (*n* = 132)	Women (*n* = 110)	Men (*n* = 115)	*p*-value
Gender of respondents (%)	100.00	36.97	30.81	32.21	
Age of respondent (years)	41.41 (11.69)	29.34 (3.63)	47.82 (8.48)	49.15 (8.60)	0.000
Relation of respondent to HHH (%)	63.59	52.27	39.09	100.00	0.000
Education level respondent (%)					
No formal education	10.36	9.85	19.09	2.61	0.000
Primary	73.11	59.09	74.55	87.83	
Secondary or higher	16.53	31.06	6.36	9.57	
Farming as the main occupation (%)	83.19	81.06	84.55	84.35	0.711
Marital status—Married (%)	86.83	92.42	69.09	97.39	0.000
Household type (%)					
Dual type	87.96	93.18	71.82	97.39	0.000
Woman only	9.52	4.55	25.45	0.00	
Man only	1.68	1.52	0.91	2.61	
Woman with absentee husband	0.84	0.76	1.82	0.00	

Standard deviation provided in parentheses

Educational attainment significantly (*p* < 0.01) differed by gender with youth farmers being highly educated (had secondary or higher education), while women farmers had the least educational qualification. 83% of respondents reported farming as their main occupation, with no significant gender differences. There were gender differences in the marital status of respondents with 69% of women being married compared to men (97%) and youths (92%). The result suggests that more female respondents were either divorced, separated, or widowed compared to men and youth respondents. This result is affirmed by significant (*p* < 0.01) differences in household types. The percentage of dual household types was higher than the percentages of women-only households (10%), men-only (2%) and women with absentee husbands (1%). These findings reveal possible differences in gender vulnerabilities to climate change and the adaptive capacity of respondents.

Table 3 shows that men significantly accessed more land (5.83 acres) than women farmers (3.78), reflecting the prevalent gender disparities in land ownership and access in Tanzania and sub-Saharan Africa. This could be attributedto cultural biases and inheritance practices favouring men over women and youth [48]. Significant differences were also observed in land ownership: 49% of men indicated that the man in the household owned the land, compared to 17% of women and 35% of youth. A higher percentage of youth (56%) reported joint land ownership (both men and women) compared to men (51%) and women (40%). Both men and women heads (53%) and female spouses (31%) demonstrated significant involvement in the management of the bean crop, confirming the common observation that common bean production is largely practised by women in sub-Saharan Africa [49].

**Table 3:** Farm characteristics and farmers’ access to institutional, technical, and social support services

Variable	Overall (N = 357)	Youths (n = 132)	Women (n = 110)	Men (n = 115)	*p*-value
Average acres of land accessed	4.38 (5.01)	3.63 (3.69)	3.78 (4.34)	5.83	0.001
Who owns the land (%)					0.000
Man	33.53	34.96	16.67	48.62	
Woman	16.47	8.13	42.59	0.00	
Both man and woman	49.41	56.10	39.81	51.38	
Other female relatives	0.29	0.81	0.00	0.00	
Rented	0.29	0.00	0.93	0.00	
Bean crop manager (%)					0.000
Both man and woman head	53.22	62.88	39.09	55.65	
Female spouse	31.09	21.97	50.91	22.61	
Male spouse	14.01	14.39	6.36	20.87	
Another household	1.68	0.76	3.64	0.87	
Average distance to agro-dealer (km)	17.52	18.33	17.65	16.47	0.541
Own mobile phones (%)	90.76	91.67	84.55	95.65	0.014
Presence on agriculture social media platform (%)	3.36	6.06	0.91	2.61	0.074
Membership to local groups/associations (%)	42.86	40.15	40.00	48.70	0.307

Standard deviation provided in parenthesis

The respondents were also asked about phone ownership to understand their accessibility to information on climate-smart farming practices. The results showed that women had marginally lower access to information via social media (3%) and mobile phones (40%) than men and youths. This happened against the backdrop of 91% of the respondents reporting that they owned mobile phones. Nonetheless, significantly more men farmers (96%) than youths (92%) and women (85%) owned a mobile phone. There were no significant gender differences in the distance to agro-dealers, with farmers having to travel for an average of 17.5 km to reach the nearest input stockists. This result suggests low access to external inputs (e.g., farm chemical) in the study areas. No significant differences were also reported for membership to local groups and associations, with about 43% belonging to groups.

Farmers affected by production challenges intimated that they responded by using pesticides to curb pests and diseases (Table 4). Farmers also used fertilizers and changed bean varieties planted. Some farmers started planting early to take advantage of the rains, while others started practising conservation agriculture and early harvesting (Table 3).

**Table 4:** Changes made in response to production constraints by gender

Change made	Total	Youths	Women	Men
Pesticide	49.39	50.00	52.73	49.09
Use fertilizer	21.34	25.00	16.36	18.18
Change variety/improved	16.46	13.89	14.55	18.18
Early/timely planting	6.10	5.56	7.27	7.27
Conservation agriculture	3.66	4.17	5.45	3.64
Sell assets	0.61		1.82	
Timely harvesting	0.61		1.82	
None	1.83	1.39		3.64

**Fig. 2:**
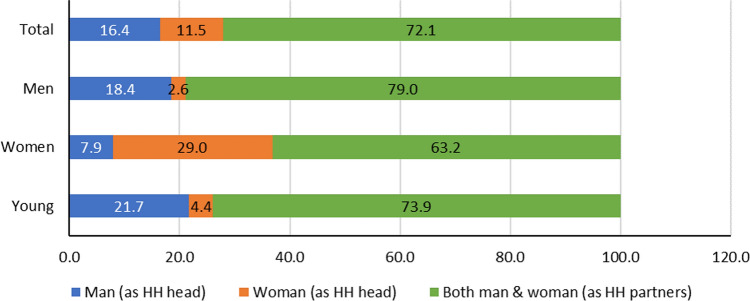
Decision maker about changes to protect bean production against production constraints by gender

The chi-square test of independence showed that there were significant gender differences (*p* < 0.01) in terms of who made decisions to respond to production constraints among affected climate change related events. More men farmers (79%) than youths (73%) and women (74%) said that both man and woman in the household made the decision to make changes in response to the production constraints (Fig. 2). The results show that a significantly higher percentage of youths indicated that men as household heads (22%) made decision about how to respond to climate change-related constraints compared to women (4.4%) and both men and women household heads (8%). In contrast, more women farmers (29%) than men (2.6%) and young farmers (4%) said that women as household heads made the decision to respond to effects of climate change. The high percentage of man and woman making joint decision could be attributed agriculture being the main occupation and gender trainings provided by interventions in common bean value chain in the two districts.

National, regional, and global development agendas and policies have earmarked climate-smart agriculture as the most appropriate response to the adverse effects of climate change. Climate-smart agriculture is a farming practice that increases the adaptive capacity of farmers and mitigates the adverse effects of climate change, as well as sustainably intensifying agricultural production [50]. Therefore, farmers were asked to report the climate-smart agricultural practices they implemented on their farms. Farmers used these climate-smart agricultural practices as a strategy to reduce climate change-related risks like drought, pests and diseases, erratic rainfall, and floods for improved production. Results in Table 5 show that conservation agriculture (62%) and pesticide use (40%) were the most frequently used climate-smart agricultural practices, followed by the use of fertilizer (30%) and improved seed (24%). However, there were no significant differences in the use of climate-smart technologies and practices by gender.

**Table 5:** Use of climate-smart agricultural technologies/practices by gender

Practice	Total	Youths	Women	Men	*p*-value
Conservation agriculture	62.46	61.36	67.27	59.13	0.428
Pesticide use	39.78	39.39	38.18	41.74	0.857
Fertilizer use	29.97	31.06	29.09	29.57	0.940
Use of improved seed	23.53	20.45	20.00	30.43	0.105

### Econometric results—multivariate probit model

The study conceptualized that men’s greater access to land and financial resources correlates with higher adoption of cost-intensive CSA practices, including fertilizer application. By contrast, women’s limited land access often leads them to adopt less capital-intensive practices such as conservation agriculture. Educational attainment, which positively impacts technology uptake, is another critical variable. Farmers with higher education levels demonstrate greater engagement with improved seed varieties and conservation agriculture due to enhanced access to agricultural information and decision-making capacity. This study controls for external factors, such as regional differences and institutional support, by including variables like distance to agro-dealers and group membership. This helps isolate the effect of individual factors on CSA adoption.

Table 6 presents multivariate probit coefficient estimates of factors that conditioned farmers’ use of climate-smart technologies and practices. The likelihood ratio test (chi^2^(60) = 272.935 Prob > chi^2^ = 0.0000) of the independence of the error terms of the different climate-smart agriculture equations was strongly rejected. The correlation coefficients of the relationship between error terms of the four equations were all positive and significant, indicating that the climate-smart technology options were complementary. Eight out of the 15 variables included in the model significantly influenced the use of improved seed, while 6, 9, and 2 variables were significantly associated with pesticides, fertilizer, and conservation agriculture (CA), respectively.

**Table 6:** Multivariate probit coefficient estimates of determinants of farmers’ use of climate-smart technologies and practices

Variable	Improved seed		Pesticides use		Fertilizer		Conservation Agric	
	Coeff	Std Err	Coeff	Std Err	Coeff	Std Err	Coeff	Std Err
Men	0.370	0.273	0.036	0.271	0.224	0.274	−0.116	0.288
Youths	0.557**	0.277	−0.096	0.264	0.592**	0.280	−0.114	0.303
Age	0.026***	0.010	−0.007	0.011	0.016	0.011	−0.001	0.011
Marital status (married)	−0.546*	0.302	−0.152	0.280	−0.623**	0.294	−0.580***	0.315
Relation to HH head (= head)	−0.125	0.232	−0.133	0.223	−0.536**	0.225	−0.205	0.241
Education level	0.393**	0.159	0.417***	0.157	0.459***	0.149	0.111	0.152
Occupation of HH head (= Farming)	1.013***	0.270	0.666***	0.216	0.727***	0.227	1.709***	0.247
Land size accessed	0.031	0.019	0.031*	0.018	0.048***	0.017	0.024	0.016
Landowner (man)	−0.224	0.192	−0.028	0.172	−0.181	0.171	−0.007	0.182
Manager of the bean plot	0.216	0.183	−0.024	0.169	0.067	0.168	0.605***	0.177
Distance to agro-dealer	−0.017***	0.007	−0.019***	0.007	−0.022***	0.007	−0.024***	0.007
Cell phone ownership	1.222***	0.429	0.443*	0.267	0.569**	0.259	−0.244	0.268
Agric social media group member	0.257	0.399	0.273	0.461	0.029	0.409	0.283	0.42
Group membership	0.766***	0.171	0.345**	0.161	0.488***	0.168	0.261	0.164
Region (=Songwe)	0.008	0.163	0.002	0.157	0.022	0.159	−0.488***	0.17
Constant	−4.402	0.835	−1.069	0.688	−2.304***	0.764	0.413	0.748
Wald chi^2^(60)	246.79***							
Likelihood ratio test	272.935***							
rho21	0.732***							
rho31	0.840***							
rho41	0.200*							
rho32	0.877***							
rho42	0.285***							
rho43	0.273***							

*, **, and *** denotes significant at 1, 5 and 10% level

Regression results in Table 6 show that youths were more likely to use improved bean seed (β = 0.557; *p* < 0.05) and fertilizer (β = 0.592; *p* < 0.05) than women farmers. These results could be explained by significantly higher access to digital information among the youth. Younger farmers learn and adapt faster to emerging technologies disseminated digitally. They connect quickly in the digital space accessing more agricultural information than older farmers. Youths also have a positive attitude towards the use of technology, making it easier for them to access and use climate-smart technologies and practices compared to older farmers, especially women. Mishra et al. [51] explained that young farmers have a positive attitude toward climate-smart technology and practices and are more likely to make farm-level changes compared to older farmers. Similar results were also reported by Kamau et al. [52] in Kenya showing that  younger farmers had a higher likelihood of using soil fertility management practices than older farmers. Murendo et al. [53] found that older farmers used manure application more than younger farmers in Zimbabwe. However, age had highly significant positive association (β = 0.026; p < 0.01) with use of improved common bean varieties. In this case, age is a proxy for farming experience, suggesting that more experienced farmers were more likely to use improved seeds than less experienced farmers. A farmer being a man and old implies that there is a likelihood of accumulated knowledge and capital which women farmers and youth lack to acquire improved seeds (Table 6).

Being married was found to have a negative influence on farmers’ use of climate-smart technologies and practices, especially in the use of improved seed (β = 0.546; *p* < 0.1), fertilizer (β = 0.623; *p* < 0.05), and conservation agriculture (β = 0.580; *p* < 0.01). This result could be attributed to various reasons, for instance, married farmers may have different priorities or financial constraints than unmarried farmers. For example, they may have more family responsibilities that require their time and resources, making it difficult to invest in new technologies. Additionally, married farmers may be more risk-averse and less willing to try new technologies, particularly if they have dependents who rely on their income from farming [54]. In contrast to Khonza et al. [55] found that married women were more likely to use fertilizers than single women. Thus, the findings in this study and other studies confirm the intersecting role of marital status in the adoption of climate-smart technologies.

The relationship (being spouse) to the household head was negatively and significantly associated with the probability of farmers using fertilizer (Table 6). Fertilizer costs form a significant proportion of costs that farmers incur in agriculture and are often subjected to price hikes, compelling farmers to make upfront purchases before seasons begin. Therefore, the acquisition of fertilizer could have represented a direct implication on household income which requires approval from  the household head as the principal decision-makers and, in most cases, are income earners and  control the use of natural and financial resources. Most households were male-headed and therefore the result indicates the importance of household headship in influencing adaptation to climate change. The results are consistent with the findings of Mwinkom et al. [56] who found that household headship had a significant influence on climate change adaptation strategies.

Having a higher education level significantly increases the likelihood of farmers adopting improved seeds (β = 0.393; *p* < 0.05), fertilizers (β = 0.417; *p* < 0.01), and pesticides (β = 0.459; *p* < 0.01). The results demonstrate that education enhanced farmers’ ability to obtain, decode and understand information. Kreft et al. [57] state that educated farmers have adequate knowledge about farm inputs such as fertilizer and pesticides and are therefore aware of the inputs to adopt in response to climate change.  As with many climate challenges, education plays a critical role in promoting awareness and expanding farmers’ social networks where they exchange knowledge about climate-smart technologies and practices. Additionally, Mlenga and Maseko [58] further affirmed that education influences the adoption of conservation technologies and practices among smallholder farmers in Eswatini. Therefore, in households where the household head or the spouse has some level of education then there are high chances of the household adopting conservation agriculture. Therefore, results suggest that the level of education contributes to the adoption of climate-smart agriculture among men, women, and youth.

Furthermore, farming as the main occupation significantly increased the likelihood of farmers adopting improved seed, pesticides use, fertilizer, and conservation agriculture (Table 6). This result was expected because having farming as the main source of livelihood possibly provided the needed motivation and incentive to farmers to invest in climates-smart agriculture that would improve adaptive capacity and resilience of farming systems to the adverse effects of climate change, thereby safeguarding household sources of food, income, and livelihoods. Obi and Maya [34] also found that farming as the main occupation is an important driver of the adoption of organic manure as well as other climate-smart agricultural practices such as crop rotation and diversification. Hasan et al. [59] found that Bangladeshi farmers whose primary occupation was agriculture were more likely to adopt climate-smart technologies and practices, reporting that the use of organic fertilizer — driven by full-time engagement in farming — was a key factor contributing to household food security.

Distance to the nearest agro dealer had a significant negative influence on farmers’ use of improved seed, pesticides, fertilizer, and conservation agriculture. Longer distance to the agro-dealer reduced the probability of using improved seed, pesticides, fertilizer, and conservation agriculture by 1.7, 1.9, 2.2, and 2.4%, respectively (Table 6). The further the farms from the main input suppliers the higher the cost of inputs and transaction costs to accessing seed, pesticides, and fertilizer. This could have discouraged the adoption of these inputs. In addition, longer distances to agro-dealers possibly limited farmers’ access to information about the role of improved seed and farm chemicals in improving the adaptive capacity of farming systems. The distance between agro-dealers and farmers, therefore, determines rural-based smallholder farmers’ access. Almekinders et al. [60] explained that agro-dealers play a critical role in guaranteeing that farmers have access to essential agricultural inputs, thereby increasing adoption rates of climate-smart technologies and practices.

Ownership of communication assets—mobile phones—also increased the chances of farmers using improved seeds, fertilizer, and pesticides (Table 6). Farmers who owned mobile phones possibly received information about available agricultural technologies for securing farming systems against climatic shocks, thereby increasing the chances of adopting climate-smart technologies. Weather and climatic information services are increasingly generating information about weather forecasts and advisory on planting and adaptation measures.  Mobile phones have also become critical channels for disseminating extension and advisory services via text and recorded voice messages which are critical to overcoming spatial constraints to access to information by farmers [61].

Group membership positively and significantly influenced the probability of farmers using improved seed, pesticides, and fertilizer by 0.77, 0.35, and 0.57, respectively (Table 6). According to Aidoo and Fromm [62] farmer associations enhance farmers’ social capital and capacity by encouraging learning. In this study, farmer groups could have created stronger social ties which possibly increased access to information about climate change, enabled innovation and technology transfer, and acted as agents of change in the climate change discourse, thereby resulting in the adoption of climate-smart agriculture. Joint decision-making increased the likelihood of using conservation agriculture by 0.61, suggesting that inputs from both household heads and spouses enhanced the use of climate-smart practices (Table 6).

Joint decision-making possibly allowed farmers to recognize the critical role of climate-smart practices in realizing the farming objectives of the household. Decisions made jointly are also more likely to be implemented because of the alignment of personal and household objectives with respect to farming. District, which captured the location of the household in Southern Highlands, was negatively associated with the chances of farmers using conservation agriculture. Farmers in Songwe district were 0.49 times less likely to use conservation agriculture compared to those in Mbeya rural, signalling the role of agro-ecological and institutional differences in the use of climate-smart practices.

## Conclusion

This study demonstrates that adoption of climate-smart agriculture CSA among smallholder farmers in Tanzania is shaped by a complex interplay of demographic and household characteristics and various forms of capital, including human, physical, social, and natural  factors. Men’s greater access to land (natural capital) and digital tools (physical capital) correlates with higher adoption of resource-intensive CSA practices like fertilizer application, while women, with more limited access to these resources, tend to adopt lower-cost options such as conservation agriculture. Youth farmers, meanwhile, benefit from educational attainment and digital literacy, enabling greater engagement with improved seed varieties and integrated pest management. These findings emphasize the critical role of educational attainment in CSA adoption, as well as the limitations that restricted access to resources impose on women and other marginalized groups. While digital tools and social networks are valuable channels for CSA knowledge dissemination, women face specific barriers including lower mobile ownership and limited digital literacy that restrict their access to these resources. Addressing these gender disparities requires targeted interventions, such as providing digital literacy training for women, increasing women’s access to mobile technology, and promoting women’s leadership within local farming cooperatives. Additionally, cultural norms that favour male land ownership and decision-making remain significant barriers to equitable CSA adoption, pointing to the need for community-level efforts to support women’s rights to land and resource access and gender-transformative approaches that tackle the deep-rooted patriarchal structures.

The environmental implications of CSA adoption are significant: conservation agriculture improves soil health; improved seed contribute to biodiversity environmental protection and water conservation. These ecological benefits align CSA adoption with Sustainable Development Goals (SDGs), particularly SDG 13 (Climate Action) and SDG 15 (Life on Land). To realize the full potential of CSA in building resilience to climate change in Tanzania, policies must address gender-specific and demographic barriers. Future research could strengthen these findings by exploring causality through longitudinal data and expanding the scope of demographic variables. Ultimately, fostering inclusive access to CSA practices will require tailored support that acknowledges the unique needs and contributions of men, women, and youth within Tanzania’s smallholder farming communities.

## Data Availability

This study’s ethical review and approval were waived since it was conducted by a government entity.

## Data availability

Data is available on request from the authors.
